# Innovations and Insights in Probiotics: Their Applications, Mechanisms, and Safety

**DOI:** 10.3390/foods15152594

**Published:** 2026-07-24

**Authors:** Mohan Li, Xiqing Yue, Zhenmin Liu

**Affiliations:** 1 College of Food Science, Shenyang Agricultural University, Shenyang 110866, China; limohan@syau.edu.cn (M.L.);; 2 State Key Laboratory of Dairy Biotechnology, Shanghai Engineering Research Center of Dairy Biotechnology, Shanghai 200436, China; 3 School of Life Sciences & Biotechnology, Shanghai Jiao Tong University, Shanghai 200240, China

In recent years, probiotics and their fundamental role in human health and food science have commanded extensive attention from both the global scientific community and the industrial sector [1,2,3]. Contemporary scientific research has definitively established that the massive microbial community colonizing the human gastrointestinal tract is not merely an accessory to the digestive system, but rather a critical physiological component deeply involved in host immune regulation, metabolic homeostasis, and neuroendocrine interaction [2,4,5,6]. Within this context, probiotics—defined as live microorganisms that confer a health benefit on the host when administered in adequate amounts—have expanded their research dimensions from early applications in food preservation and basic fermentation to the advanced stages of targeted disease intervention and precise physiological modulation [7,8,9,10,11,12]. Studies have found that different strains demonstrate highly significant variances in their survival capacity within the gastrointestinal environment, their mechanisms of colonization and adhesion to the intestinal mucosa, and the secondary metabolites they produce [13,14,15,16]. Concurrently, with the continuous global escalation in the incidence rates of chronic metabolic diseases, neurodegenerative disorders, and psychiatric conditions, the medical field requires novel targeted therapeutic strategies capable of intervening in disease progression through non-invasive modalities [17,18,19,20]. This Special Issue “Innovations and Insights in Probiotics: Their Applications, Mechanisms, and Safety” was established on this scientific background, with the objective of comprehensively presenting the latest breakthroughs in this specific discipline.

This Special Issue aims to construct a comprehensive academic platform encompassing probiotic application innovation, functional mechanism elucidation, processing technology advancement, and safety evaluation through rigorous scientific investigation. Although the application of probiotics in various fermented foods possesses a long history, maximizing their viability during processing, storage, and gastrointestinal delivery via advanced processing technologies—such as microencapsulation and targeted controlled release systems—remains a major engineering challenge for the food and pharmaceutical industries [21,22,23,24]. More fundamentally, elucidating the molecular and cellular mechanisms by which probiotics exert their health benefits is a mandatory prerequisite for facilitating their transition from traditional nutritional supplements to high-tier clinical therapeutics. This necessitates a detailed analysis of how probiotics execute systemic pathological regulation of the host through microscopic pathways, including the secretion of short-chain fatty acids, the modulation of tight junction protein expression in intestinal epithelial cells, and intervention in the host’s hypothalamic–pituitary–adrenal axis. Furthermore, with the widespread implementation of high-throughput sequencing technologies and bioinformatics analysis, safety evaluation standards for probiotics have strictly advanced from traditional phenotypic testing to the whole-genome level [7,25,26]. This requires researchers to thoroughly examine whether candidate strains carry transferable antibiotic resistance genes or potential virulence factors without omission. The six articles included in this Special Issue stem from advanced scientific concepts such as next-generation probiotics, postbiotics, and the complex gut–brain axis, providing robust evidence to elucidate the intricate physiological interactions between microorganisms and host health.

Regarding strict safety evaluation and the discovery of strain-specific functions, the first article in this Special Issue conducted an exhaustive whole-genome sequencing and in vitro characterization of the *Bifidobacterium animalis* subsp. *lactis* BD1 strain sourced from infant feces. In-depth comparative analyses utilizing the Virulence Factor Database demonstrated that the BD1 strain contains only 139 low-similarity homologs, and no major toxin genes were detected. Addressing the critical global public health concern of antibiotic resistance, the study confirmed that the BD1 genome contains exactly four chromosomally encoded resistance genes. In particular, although the strain carries the tetracycline resistance gene tet(W) within the genomic island GI01, the total absence of associated mobile genetic elements in its vicinity renders the risk of horizontal gene transfer negligible. On a metabolic functional level, the BD1 strain exhibits a strong capacity for carbohydrate and amino acid metabolism, possessing a genome rich in carbohydrate-active enzymes that ensure the efficient degradation and utilization of starch, sucrose, and galactose. Additionally, the significant enrichment of lipid metabolism pathways, such as glycerolipid and sphingolipid metabolism, indicates its substantial development potential in enhancing fermented food profiles and regulating host lipid metabolism. Combined with its strong bile salt tolerance, cell surface hydrophobicity, and biofilm-forming capacity, this study rigorously verified that BD1 possesses the exact characteristics required for development as a novel functional probiotic strain.

The authors of the second article in this Special Issue examined emerging neurotherapeutic strategies involving probiotics and the gut–brain axis in the comorbidity of epilepsy and depression. Precise epidemiological data indicate that the prevalence of depression in patients with epilepsy reaches 20 to 55 percent, while depression simultaneously increases the risk of developing epilepsy by a factor of 2.5. The review systematically elucidated the underlying bidirectional pathophysiological mechanisms shared between these two destructive disorders, specifying that their common neurobiological root lies in persistent central neuroinflammation, hypothalamic–pituitary–adrenal axis dysregulation, and severe glutamatergic system dysfunction. Neuroinflammatory mediators, including interleukin-6, tumor necrosis factor-alpha, and high-mobility group box 1, establish a pathological cycle within the patient’s brain: epileptic seizures directly exacerbate local neuroinflammation and mood dysregulation, while chronic psychological stress subsequently lowers the seizure threshold via physiological pathways. Facing this complex neural network damage, the authors proposed novel therapeutic interventions targeting these shared mechanisms. Probiotics demonstrate an exceptional capacity to repair glial cell function and mitigate central nervous system degenerative inflammation by precisely manipulating the gut–brain axis, particularly by promoting the massive synthesis of neuroprotective metabolites such as butyrate by intestinal anaerobic microorganisms. The authors asserted that by integrating epigenome editing technologies such as CRISPR and biomarker-guided precision medicine, the medical community must complete a fundamental clinical treatment paradigm shift from the mere suppression of seizure symptoms to systemic mechanistic repair.

In the broader context of neurodegenerative disease prevention, the third article in this Special Issue provided a comprehensive and systematic review of the critical role of probiotics in modulating the gut microbiome pathological mechanisms in Alzheimer’s disease. Alzheimer’s disease has emerged as a global public health crisis threatening the aging population; its pathogenesis is exceptionally complex, and the clinical efficacy of existing synthetic chemical drugs remains highly limited. The authors detailed how intestinal microecological dysbiosis compromises the physical density of the intestinal epithelial barrier, leading to the leakage of pro-inflammatory substances such as endotoxins into the systemic circulation. This process causes the abnormal activation of peripheral immune responses mediated by microbial metabolites, ultimately destroying the absolute permeability restrictions of the blood–brain barrier. This systemic immune inflammation, spreading from the digestive tract to the central nervous system, drastically accelerates the programmed apoptosis of brain neurons. The review demonstrated the potential of probiotic interventions: by competitively excluding pathogenic bacteria, repairing damaged intestinal mucosal tight junctions, and directionally remodeling the microbial metabolome profile, specific probiotics can efficiently block the generation and cross-system transmission of pathogenic inflammatory signals from their intestinal source, thereby establishing an irrefutable scientific rationale for developing novel clinical intervention strategies for Alzheimer’s disease based on microecological reconstruction.

As the understanding of the interaction mechanisms between microbial entities and hosts continues to refine, postbiotics—comprising inactivated probiotic cells and their complex metabolites—have demonstrated massive translational medical value. The fourth article in this Special Issue presented a highly prospective experimental study that evaluated the molecular efficacy of postbiotics derived from *Limosilactobacillus fermentum* and *Lactobacillus delbrueckii* subsp. *lactis* in protecting the intestinal barrier function. Within this three-dimensional system, which allows for 15 days of continuous culture and accurately replicates human colonic microecological dynamics, researchers utilized afatinib to precisely induce the pathological disintegration of the epithelial barrier, subsequently introducing postbiotic interventions at gradient doses of 5, 10, or 20 mg/mL. The physical measurement results conclusively demonstrated that all concentrations of postbiotics significantly promoted the rapid recovery of trans-epithelial electrical resistance—the core physical indicator of epithelial integrity—during the late stages of pathological damage. More importantly for clinical application, the high-dose intervention of 20 mg/mL exhibited absolute suppressive efficacy against a series of core pro-inflammatory chemokines and cytokines, including CCL2, CX3CL1, CXCL1, CXCL5, IL-8, and IL-11. This research not only confirms the dose-dependent principle of specific postbiotics in driving a damaged intestinal microenvironment toward an anti-inflammatory phenotype but also demonstrates the immense potential of organ-on-a-chip technology in screening and evaluating novel microecological preparations.

The fifth article in this Special Issue focused on *Akkermansia muciniphila*, a hallmark strain of next-generation probiotics currently commanding global scientific attention, to systematically analyze its molecular target roles in preventing and intercepting the progression of diabetes. *Akkermansia muciniphila* is an obligate Gram-negative anaerobic bacterium colonizing the intestinal mucus layer; the article addressed a critical clinical point by explicitly identifying the strict pathological correlation between the onset of diabetes, its complex complications, and the catastrophic depletion of *Akkermansia muciniphila* abundance in the intestine. The researchers detailed how core components of *Akkermansia muciniphila*—including the outer membrane protein Amuc_1100, bacteria-derived extracellular vesicles and specific secreted proteins P9 and Amuc_1409—systematically repair the damaged intestinal mucosal barrier and profoundly intervene in systemic insulin sensitivity through specific binding with host intestinal epithelial receptors. Simultaneously, the article comprehensively cataloged current targeted dietary interventions and pharmacological regulatory methods developed to alter *Akkermansia muciniphila* abundance. Through detailed molecular mechanism summarization, this review not only established the scientific status of *Akkermansia muciniphila* as a critical target for metabolic correction in diabetes but also outlined a clear technical trajectory to overcome its obligate anaerobic cultivation bottlenecks and achieve large-scale pharmaceutical development in the future.

Regarding the direct physiological intervention applications for widespread metabolic diseases, the sixth article in this Special Issue presented a review demonstrating the application value of probiotic supplementation in remodeling the body’s blood glucose regulation mechanisms and treating type 2 diabetes mellitus. Driven by the consolidation of global industrialized dietary patterns, the incidence of diabetes induced by long-term nutritional overload constitutes a severe global medical crisis. Current routine clinical applications of glucose-lowering chemical drugs and exogenous insulin injections can forcibly alter blood biochemical indicators, but they remain completely incapable of blocking the deteriorating progression of the underlying metabolic disorder, and they are accompanied by severe side effects such as irreversible gastrointestinal damage and hypoglycemic crises. Probiotics, serving as a microecological reconstruction solution with exceptional safety that complies with human natural metabolic laws, are becoming the critical breakthrough point in this field. Mechanistic analyses indicate that targeted probiotic intake not only suppresses the proliferation of pathogenic flora but directly blocks the systemic chronic low-grade inflammation induced by enterogenous endotoxins—with systemic inflammation identified as the fundamental culprit inducing insulin resistance. Furthermore, following colonization, probiotics significantly upregulate the efficiency of intestinal endocrine cells in synthesizing and releasing glucagon-like peptide-1, thereby enhancing the glucose-dependent insulin secretion of pancreatic beta cells in a manner entirely consistent with human physiological rhythms. By showing the increase in the substrate concentration of short-chain fatty acids and the comprehensive downregulation of cellular oxidative stress damage, this review provided the most comprehensive and solid biochemical theoretical foundation for the universal application of probiotic preparations in the precise management of metabolic diseases and the development of functional foods.

In summation, this Special Issue “Innovations and Insights in Probiotics: Their Applications, Mechanisms, and Safety” makes a contribution to elevating human comprehension of probiotic biological characteristics by scientifically selecting and compiling six original research papers and systematic reviews of academic value. Looking forward, the global development of probiotic science stands at a decisive turning point, transitioning entirely from the era of crude empirical food additives to an era of precise medical targeted interventions highly dependent on precise omics data. The successful publication of this Special Issue does not represent the terminus of research, but rather a rallying call to mount an offensive against higher-level scientific challenges. Future scientific research must concentrate resources on overcoming the core technical barriers that currently restrict the industrial transformation of this field. This involves establishing personalized probiotic prescription standards based on individual baseline gut microbiota characteristics at the clinical end, developing revolutionary encapsulation and delivery materials at the engineering end to ensure next-generation extreme anaerobes maintain ultra-high viability during continuous industrial production and while crossing the gastric acid barrier, and constructing a long-cycle absolute safety evaluation system at the regulatory end specifically for genetically modified recombinant strains and complex postbiotic mixtures. It is only through the unreserved, deep, and interdisciplinary collaboration of foundational microbiologists, frontline clinical medical experts, biomedical engineering scholars, and global food and pharmaceutical conglomerates that these laboratory molecular insights—harboring disruptive therapeutic potential—can be transformed into highly efficient targeted clinical drugs and daily health intervention vehicles accessible to all of humanity.

## References

[1] Xie A.J., Zhao S.S., Liu Z.F., Yue X.Q., Shao J.H., Li M.H., Li Z.W. (2023). Polysaccharides, proteins, and their complex as microencapsulation carriers for delivery of probiotics: A review on carrier types and encapsulation techniques. Int. J. Biol. Macromol..

[2] Sarita B., Samadhan D., Hassan M.Z., Kovaleva E.G. (2025). A comprehensive review of probiotics and human health-current prospective and applications. Front. Microbiol..

[3] Sharma I., Bharti S., Pandey S., Gupta S., Bisen P.S., Kango N., Sharma K.K. (2026). Leveraging computational methodologies for probiotic research: Unraveling host-microbiota interactions and therapeutic potential. Food Nutr..

[4] Mazziotta C., Tognon M., Martini F., Torreggiani E., Rotondo J.C. (2023). Probiotics mechanism of action on immune cells and beneficial effects on human health. Cells.

[5] Shen X.Y., Xie A.J., Li Z.J., Jiang C.X., Wu J.Q., Li M.H., Yue X.Q. (2024). Research progress for probiotics regulating intestinal flora to improve functional dyspepsia: A review. Foods.

[6] Li Z.J., Kanwal R., Yue X.Q., Li M.H., Xie A.J. (2024). Polyphenols and intestinal microorganisms: A review of their interactions and effects on human health. Food Biosci..

[7] da Silva T.F., Glória R.D., Americo M.F., Freitas A.D., de Jesus L.C.L., Barroso F.A.L., Laguna J.G., Coelho-Rocha N.D., Tavares L.M., Le Loir Y. (2024). Unlocking the potential of probiotics: A comprehensive review on research, production, and regulation of probiotics. Probiotics Antimicrob. Proteins.

[8] Zhang Y.M., Xie Y.F., Shao J.H., Yue X.Q., Li M.H. (2025). Maillard reaction-based conjugates as carrier strategies for delivery of bioactive compounds: A review. Curr. Opin. Food Sci..

[9] Zhao R.A., Yu T., Li J.H., Niu R.H., Liu D.H., Wang W.J. (2024). Single-cell encapsulation systems for probiotic delivery: Armor probiotics. Adv. Colloid Interface Sci..

[10] Chandrasekaran P., Weiskirchen S., Weiskirchen R. (2024). Effects of probiotics on gut microbiota: An overview. Int. J. Mol. Sci..

[11] Santos R.O., Silva M.V.F., Nascimento K.O., Batista A.L.D., Moraes J., Andrade M.M., Andrade L., Khosravi-Darani K., Freitas M.Q., Raices R.S.L. (2018). Prebiotic flours in dairy food processing: Technological and sensory implications. Int. J. Dairy Technol..

[12] Abouelela M.E., Helmy Y.A. (2024). Next-generation probiotics as novel therapeutics for improving human health: Current trends and future perspectives. Microorganisms.

[13] Bubnov R.V., Babenko L.P., Lazarenko L.M., Mokrozub V.V., Spivak M.Y. (2018). Specific properties of probiotic strains: Relevance and benefits for the host. Epma J..

[14] Wang Y., An M.X., Lv Y.M., Hou Y.Q., Li Z.H., Dai J.H., Ni W., Hu S.W. (2026). Genomic and functional characterization of probiotic strains from traditional fermented dairy products in alleviating dextran sulfate sodium-induced colitis. J. Dairy Sci..

[15] Fentie E.G., Lim K., Jeong M., Shin J.H. (2024). A comprehensive review of the characterization, host interactions, and stabilization advancements on probiotics: Addressing the challenges in functional food diversification. Compr. Rev. Food Sci. Food Saf..

[16] Lin Q.J., Lin S.Y., Fan Z.T., Liu J., Ye D.C., Guo P.T. (2024). A review of the mechanisms of bacterial colonization of the mammal gut. Microorganisms.

[17] Capucho A.M., Chegao A., Martins F.O., Miranda H.V., Conde S.V. (2022). Dysmetabolism and neurodegeneration: Trick or treat?. Nutrients.

[18] Ney J.P., Steinmetz J.D., Anderson-Benge E., Gillespie C.W., Becker A., Steele X., Esper G.J. (2026). Us burden of disorders affecting the nervous system. JAMA Neurol..

[19] Cunnane S.C., Trushina E., Morland C., Prigione A., Casadesus G., Andrews Z.B., Beal M.F., Bergersen L.H., Brinton R.D., de la Monte S. (2020). Brain energy rescue: An emerging therapeutic concept for neurodegenerative disorders of ageing. Nat. Rev. Drug Discov..

[20] Sovrea A.S., Bosca A.B., Dronca E., Constantin A.M., Crintea A., Sufletel R., Stefan R.A., Stefan P.A., Onofrei M.M., Tschall C. (2025). Non-drug and non-invasive therapeutic options in alzheimer’s disease. Biomedicines.

[21] Xie A.J., Gao M.G., Du H.S., Pan X.J. (2025). Next-generation probiotics delivery: Innovations and applications of single-cell encapsulation. Curr. Opin. Food Sci..

[22] Xu C., Guo J.H., Chang B.Y., Zhang Y.M., Tan Z.M., Tian Z.H., Duan X.L., Ma J.G., Jiang Z.M., Hou J.C. (2024). Design of probiotic delivery systems and their therapeutic effects on targeted tissues. J. Control. Release.

[23] Li C.C., Wang Z.X., Xiao H.N., Wu F.G. (2024). Intestinal delivery of probiotics: Materials, strategies, and applications. Adv. Mater..

[24] Zhang J.R., Liu B.Q., Xie Q.G., Wang J.Y., Ma L., Yang C., Yang X.Y., Guo L., Su Y., Jiang Y.J. (2026). Polysaccharide-based microgels for probiotic delivery: Advances in design, fabrication, and functional food applications. Trends Food Sci. Technol..

[25] Parvin T., Sadras S.R. (2024). Advanced probiotics: Bioengineering and their therapeutic application. Mol. Biol. Rep..

[26] Zaghloul E.H., El Halfawy N.M. (2024). Marine pediococcus pentosaceus e3 probiotic properties, whole-genome sequence analysis, and safety assessment. Probiotics Antimicrob. Proteins.

